# Liquid–liquid phase separation in human health and diseases

**DOI:** 10.1038/s41392-021-00678-1

**Published:** 2021-08-02

**Authors:** Bin Wang, Lei Zhang, Tong Dai, Ziran Qin, Huasong Lu, Long Zhang, Fangfang Zhou

**Affiliations:** 1 Department of Orthopaedic Surgery, The Third Affiliated Hospital of Wenzhou Medical University, Rui’an, People’s Republic of China; 2 MOE Laboratory of Biosystems Homeostasis & Protection and Innovation Center for Cell Signaling Network, Life Sciences Institute, Zhejiang University, Hangzhou, People’s Republic of China; 3 Institutes of Biology and Medical Science, Soochow University, Suzhou, People’s Republic of China

**Keywords:** Diseases, Cell biology

## Abstract

Emerging evidence suggests that liquid–liquid phase separation (LLPS) represents a vital and ubiquitous phenomenon underlying the formation of membraneless organelles in eukaryotic cells (also known as biomolecular condensates or droplets). Recent studies have revealed evidences that indicate that LLPS plays a vital role in human health and diseases. In this review, we describe our current understanding of LLPS and summarize its physiological functions. We further describe the role of LLPS in the development of human diseases. Additionally, we review the recently developed methods for studying LLPS. Although LLPS research is in its infancy—but is fast-growing—it is clear that LLPS plays an essential role in the development of pathophysiological conditions. This highlights the need for an overview of the recent advances in the field to translate our current knowledge regarding LLPS into therapeutic discoveries.

## Introduction

An obvious feature of biological evolution at the cellular level is the development of diverse organelle structures from a relatively uniform cytoplasmic environment. Eukaryotic cells contain various organelles that harbor distinct chemical microenvironments, which sequester molecules and proteins to increase the reaction rates of specific processes. Eukaryotic cells harbor two kinds of organelles, i.e., (a) membrane-bound organelles, such as endoplasmic reticulum, which contain components necessary to synthesize, process, and transport secreted proteins, and (b) membraneless organelles, such as Cajal bodies that are known to play a vital role in small nuclear ribonucleoprotein assembly and ribosome biogenesis.^1–3^

The lipid bilayer membranes of the membrane-bound organelles allow the enclosure of specific proteins, nucleic acids, and other molecules within a restricted space, a phenomenon that enables the organalles to perform their functions. Leakage of these proteins or nucleic acids from the organelles might result in serious consequences; for example, the release of cytochrome c into the cytoplasm results in apoptosis, and the release of nucleic acids into the cytoplasm results in the activation of the innate immune pathway.^4,5^ In comparison, membraneless organelles do not have any encapsulating membrane to limit molecular exchange. These membraneless structures exist stably in eukaryotic cells and frequently exchange various molecules with the surrounding cytoplasm. A fundamental question in cell biology is “how are these membraneless compartments organized to control such complex biochemical reactions in space and time.”

Liquid–liquid phase separation (LLPS) is gaining acceptance as a powerful mechanism to explain the formation of membraneless organelles and their functions.^6,7^ Multivalent macromolecular interactions can drive the transition of some proteins into another phase with different physiochemical properties to induce the formation of membraneless organelles or cell structures.^6,8^ Both membraneless organelles and cell structures exhibit higher protein density and weaker molecular motion than the surrounding medium, allowing for increased rates of biochemical reactions.^6,7,9^ In most cases, these structures exhibit liquid characteristics, and are therefore described as bodies, puncta, granules, droplets, and condensates.We characterize condensates into three groups, i.e., plasma membrane, cytoplasm, and nuclear-localized condensates. These condensates play unexpected roles in various cellular processes (Table 1).

**Table 1:** Examples of the various biomolecular condensates and their functions

Localization	Condensates	Biological process	Refs
Plasma membrane	TCR clusters	T-cell immune signal transduction	^14^
	Nephrin clusters	Glomerular filtration barrier	^12,187,188^
	Actin patches	Endocytosis	^189^
	Focal adhesions	Cell adhesion and migration	^190^
	Synaptic densities	Neurotransmission	^16,17^
Cytoplasm	Stress granule	mRNA storage and translational regulation	^191,192^
	RNA transport granule	mRNA storage and transport in neuronal cells	^29^
	U body	Storage and assembly of snRNPs	^193^
	P body	mRNA decay and silencing	^192^
	Balbiani body	A transient collection of proteins, RNA, and membrane-bound organelles found in primary oocytes of all animals observed to date	^194,195^
	P granule	Germ cell lineage maintenance in *Caenorhabditis elegans*	^11^
	cGAS condensates	Innate immune signaling	^15^
Nucleus	Cleavage body	mRNA processing	^196^
	Cajal body	Assembling spliceosomal small nuclear ribonucleoproteins	^197,198^
	Gem	Aid histone mRNA processing	^199,200^
	Nuclear speckles	mRNA splicing	^201^
	OPT domain	Transcriptional regulation	^202,203^
	PcG body	Transcriptional repression	^204^
	PML bodies	Apoptotic signaling, anti-viral defense, and transcription regulation.	^205^
	Histone locus body	Processing of histone mRNAs	^206^
	Paraspeckles	Storage of certain RNAs	^207^
	Perinucleolar compartment	Related to malignancy	^208^

In this review, we briefly review the development of the LLPS concept and discuss recently developed methods for studying LLPS. We highlight important findings regarding the mechanisms underlying LLPS-modulated transcription, genome organization, immune responses and neuronal synaptic signaling. We describe the role of LLPS in human diseases, including neurodegenerative diseases, cancer, and COVID-19.

Although research on LLPS is increasing, we are beginning to understand that this phenomenon plays an essential role in physiology and disease. This highlights the need for an updated overview of the recent advances in the field to translate the existing information on LLPS into therapeutic discoveries.

## Development of the LLPS concept and methods to study this phenomenon

### A brief introduction to the development of the LLPS

**Fig. 1:**
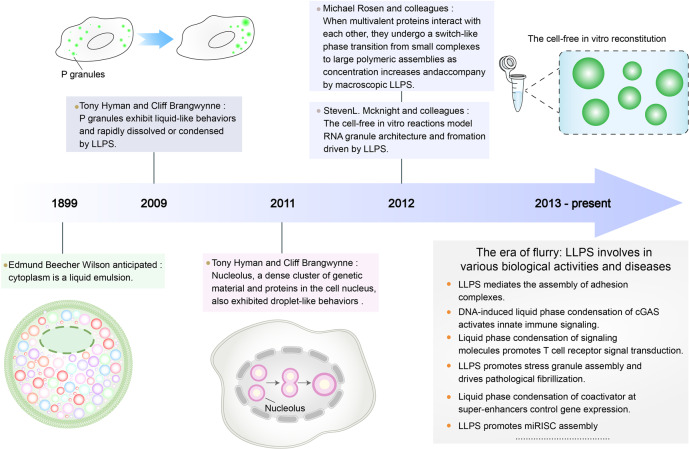
History of the discovery and development of LLPS. Representative milestone findings promoting the development of LLPS are enumerated in the figure

The hypothesis that the main bulk of a cell, the cytoplasm, resembles and behaves like a mixture of different chemically suspended drops, was proposed by Edmund Beecher Wilsonin in 1899 (Fig. 1).^10^ However, it was not until 2009—when Tony Hyman and Cliff Brangwynne found that P granules exhibit liquid-like behavior and that their localization is driven by rapid dissolution/condensation—that researchers began to realize that LLPS might underlie the organization of various membraneless organelles.^11^ In 2012, Michael Rosen and colleagues found that when multivalent proteins interact, they undergo a rapid transition from small complexes to large polymeric assemblies with increase in protein concentration and that this phenomenon is accompanied by macroscopic LLPS.^12^ In the same year, Steven L. McKnight and colleagues showed that LLPS determined the architecture of RNA granules in a cell-free in vitro reaction.^13^ The above two studies showed that phase separation was easily achieved in test tubes using simple procedures, making it easier to study LLPS. Since then, this field has expanded, with many groups studying LLPS. Emerging research has demonstrated that LLPS is involved in various processes, such as adaptive and innate immune signaling, stress granule assembly, heterochromatin formation, transcription, miRISC assembly, autophagy. LLPS has also been to play a role in the development of cancer and neurodegenerative and inflammatory diseases.^14–30^

**Fig. 2:**
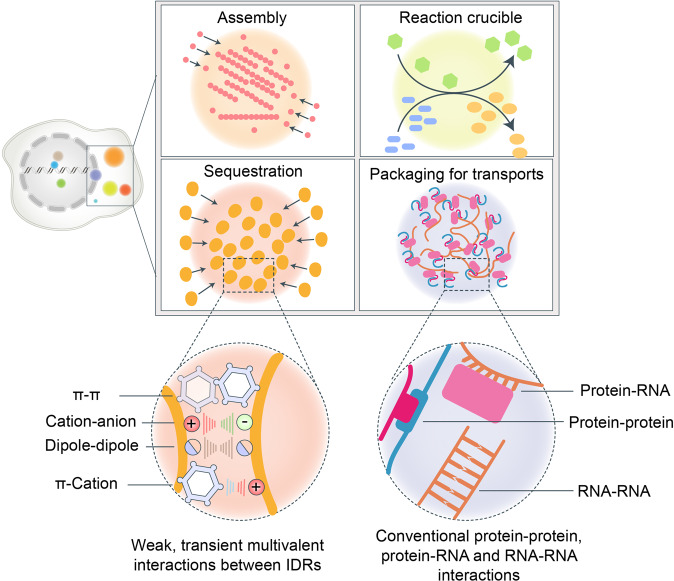
The forces driving LLPS. There are several functions of biomolecular condensates, including the assembly of a large complex, as a reaction crucible promoting biochemical reaction, sequestration of specific proteins to inhibit or promote some reaction, and packaging for transports. Besides, there are two types of multivalent interactions that contribute to LLPS. One is conventional multivalent interactions between protein and protein, protein and RNA, or RNA and RNA. The other is weak, transient, multivalent interactions between intrinsically disordered regions (IDRs), including π–π interactions, cation–anion interactions, dipole–dipole interactions, and π–cation interactions

Studies uncover that two types of multivalent interactions contribute to LLPS (Fig. 2), i.e., intracellular protein-protein, protein–RNA, and RNA–RNA interactions and weak, transient, multivalent interactions between intrinsically disordered regions (IDRs), including the π–π interactions, cation–anion interactions, dipole–dipole interactions, and π–cation interactions.^8,12,31^ LPPS can be described as a product of the force of electrochemical gradients within cells, and these gradients are established by the multivalent interactions, which influence and are influenced by the spatial arrangement of molecules in the droplets.

### Methods to identify and study liquid separation

As mentioned earlier, emerging evidence indicates the involvement of LLPS in various cellular processes. Methods to study liquid separation are being developed (to observe LLPS); we review them below.

**Fig. 3:**
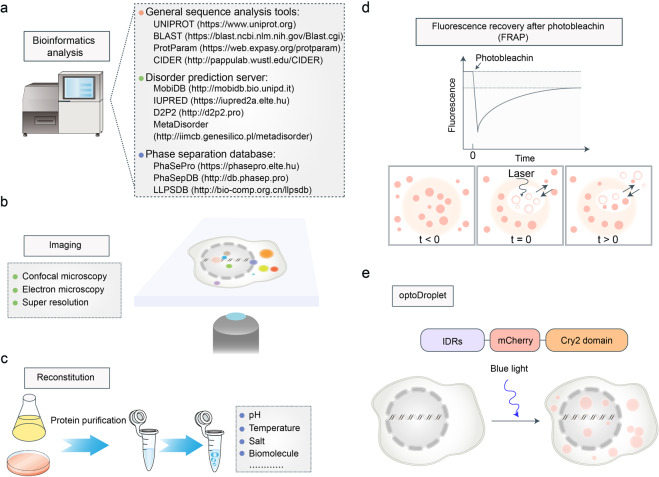
The methods to identify or study LLPS. **a** Selected bioinformatic tools or databases for studying LLPS. **b** Electron microscopy, confocal microscopy, and super-resolution imaging techniques can provide detailed information on biomolecular condensates. **c** The cell-free reconstitution assay can detect the specific phase separation conditions of targeted proteins in vitro. **d** Fluorescence recovery after photobleaching can detect the material properties and dynamics of biomolecular condensates. After being photobleached by laser, the fluorescence of condensates will recover over time. The less time condensates take to recover, the higher is their fluidity. **e** OptoDroplet system can regulate multivalency using blue light to promote or reverse the formation of biomolecular condensates in vivo. Cry2, an Arabidopsis thaliana protein domain that forms oligomers following blue-light activation, are fused to the IDRs from targeted proteins and fluorescently tagged proteins

Several tools have been developed to predict the ability of targeted proteins to undergo phase separation (Fig. 3a). For example, D2P2 is a database that curates predictions on disorder and binding sites, information regarding protein family domains, and post-translational modifications. In addition to disorder prediction, several phase separation databases provide information regarding experimentally verified cases of LLPS, integrating a wide range of information on biophysical driving forces, biological function, and regulation of these molecular systems. These analysis tools or databases can help us to quickly predict or analyze the phase separation abilities of targeted proteins.

Microscopy is an essential tool to visualize the structure and composition of these biomolecular condensates (Fig. 3b). Recent advances in confocal microscopy and super-resolution imaging provide more detailed information on biomolecular condensates. For example, the core-shell architecture of the SARS-CoV-2 N protein + RNA + M protein condensates was revealed using super-resolution imaging.^32^ However, the disadvantage of these two techniques is that the condensates must be antibody-stained or fluorescently labeled. Electron microscopy can also be used to precisely visualize the biomolecular condensates in a label-free manner.^33^ The combination of microscopy and in vitro reconstitution assays is widely used for studying LLPS. Microscopy can be used to study the phase separation of proteins purified from *E. coli*, yeast, or insect cells (Fig. 3c). LLPS is sensitive to some factors, i.e., pH, temperature, RNA, salt concentrations, and post-translational modifications.^34–37^ Therefore, we can identify the specific phase separation conditions for target proteins in vitro.

The material properties and dynamics of biomolecular condensates are determinants of their functions. For example, the gel-like N protein condensates promote nucleocapsid assembly, whereas the liquid-like N protein condensates promote viral genome processing.^38^ The material properties and dynamics of biomolecular condensates can be investigated using imaging-based techniques, including fluorescence recovery after photobleaching (FRAP), fluorescence loss in photobleaching (FLIP), and fluorescence correlation spectroscopy (FCS).^39^ Among these techniques, FRAP is the most extensively used. After being photobleached by laser, the fluorescence of condensates recovers over time (Fig. 3d). The less time the condensates take to recover, higher the fluidity.

Although in vitro studies aid the investigation of the properties of biomolecular condensates, the in vivo composition, concentration, and function of these biomolecular condensates are not well understood. Currently, an optogenetics-based system, which can regulate multivalency using blue light to promote or reverse the formation of biomolecular condensates in vivo, has been developed (Fig. 3e).^40^ This system is called optoDroplet. Cry2, an *Arabidopsis thaliana* protein domain, that forms oligomers following blue-light activation, is fused to the IDRs from target proteins and is fluorescently tagged. We can use the optoDroplet system to study the role of condensates in promoting biological function or dysfunction in vivo, which is difficult with other methods. Overall, these novel methods can provide insights into the mechanisms of action and functions of LLPS.

## Physiological functions of LLPS

### LLPS regulates transcription

Transcription is an essential part of gene expression. Abnormal regulation of transcription could lead to disease development.^41^ Therefore, transcription is strictly regulated by various factors. In the past few decades, genetics, biochemistry, molecular biology, and genomic studies have identified numerous factors, such as, transcription factors, coactivators, and enhancers that play an essential role in regulating transcription.^42–44^ However, there is little information as to how these factors work together to establish complex regulatory process. Recent studies have shown that LLPS is a new regulatory mechanism that these factors use to control transcription.^21,45–51^

**Fig. 4:**
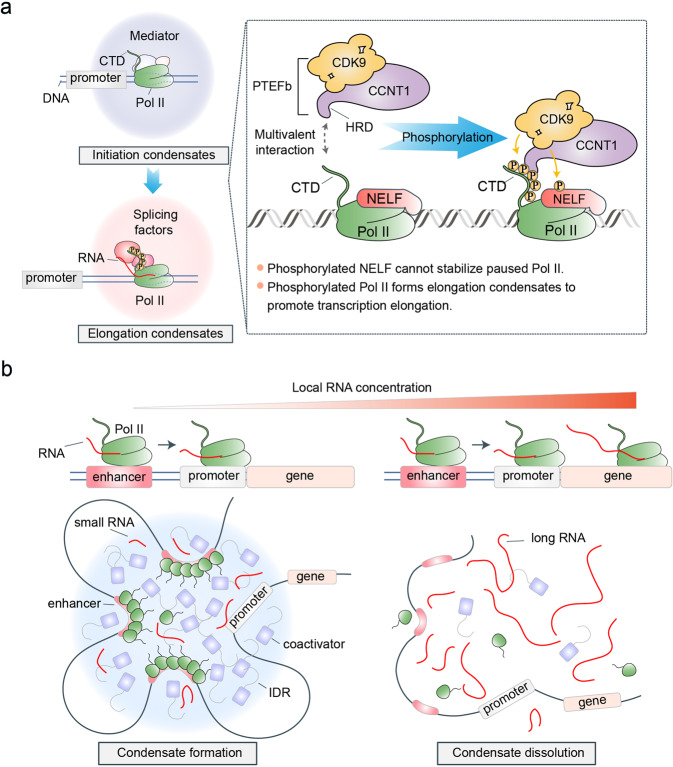
The function of LLPS in regulating gene transcription. **a** Phosphorylation regulates the transformation of initiation condensates to elongation condensates. In transcriptional initiation, RNA polymerase II (Pol II) phase-separates various factors with IDRs, such as transcription factors and coactivators, to form initiation condensates. After the transcription initiation, Pol II does not directly enter the elongation phase but pauses in a region approximately 50 bp downstream of the transcription start site, which is called promoter-proximal pausing. Thereafter, PTEFb can phase-separate into the initiation condensates through the multivalent interactions between the histidine-rich domain and carboxy-terminal domain (CTD) of Pol II. Therefore, CDK9, a subunit domain of PTEFb, can phosphorylate the negative elongation factor (NELF) and CTD. Phosphorylated NELF cannot stabilize paused Pol II and phosphorylated Pol II forms elongation condensates by hyperphosphorylated CTD, thereby achieving transcription elongation. **b** The transcriptional condensates can also be formed at a super-enhancer. Besides, the local RNA concentration can negatively regulate the formation of super-enhancer condensates. Low levels of RNA at regulatory DNA elements promote the formation of transcriptional condensates, whereas high levels of RNA from gene transcription can dissolve the transcriptional condensates

The enzyme, RNA polymerase (Pol) uses DNA as a template to catalyze the synthesis of RNA. Until now, three eukaryotic RNA polymerases have been discovered (Pol I, Pol II, Pol III).^52^ Pol I catalyzes the transcription of the large ribosomal RNA precursor, Pol III synthesizes tRNAs and the small rRNA, and Pol II—considered the most important of the RNA polymerases—transcribes mRNAs and a variety of non-coding RNA.^53^ Live-cell super-resolution microscopy has revealed that Pol-II-mediated transcription takes place at nuclear condensates, which are also called clusters, hubs, or foci.^21,46,47,54–56^ These nuclear condensates exhibit properties of a liquid, i.e., rapid recovery of fluorescence after photobleaching and sensitivity to 1,6-hexanediol, a hydrophobic compound that impairs phase separation. These nuclear condensates are formed by the LLPS of Pol II and various factors harboring IDRs, such as transcription factors and coactivators.^48^ Interestingly, these nuclear condensates are not only found at the promoter, but also at the super enhancers, which consists of a cluster of enhancers (Fig. 4b).^21^ Super enhancers have a higher density of transcription factors and coactivators than typical enhancers and function to activate the transcription of the key genes that determine cell identity.^57,58^ This is achieved by the phase separation of transcription factors and coactivators, a process driven in part by weak and transient multivalent interactions of the IDRs.^21,48^ Transcription factors usually contain one or more transactivation domains with a disordered structure and one or more DNA-binding domains with a specific structure.^59^ Recent advances in phase separation indicate that diverse transcription factors, including the embryonic stem cell pluripotency transcription factor OCT4, the ligand-dependent transcription factor estrogen receptor, and the yeast transcription factor GCN4, can form phase-separated condensates with Mediator, a coactivator complex. Mediator can stabilize the preinitiation complexes and promote transcription, to recruit the intrinsically disordered carboxy-terminal domain (CTD) of Pol II, thereby initiating the transcription of the target gene.^48,60^ Additionally, Boehning et al. found that the CTD of Pol II alone can form condensates in the presence of molecular crowding agents.^47^ This may indicate that the CTD is a target of transcription condensates, which allows Pol II to be recruited to active genes.

There are three key steps in transcription, i.e., initiation, elongation, and termination. After the initiation of transcription, Pol II does not directly enter the elongation phase but pauses in a region approximately 50 bp downstream of the transcription start site, a phenomenon called promoter-proximal pausing.^61–63^ As a key rate-limiting step in transcription and an important early checkpoint, promoter-proximal pausing must ensure that the correct modification of Pol II and the capping of the 5′ of the nascent RNA are transcribed before Pol II enters the elongation phase.^64^ Recent progress in understanding LLPS indicates that the transition from promoter-proximal pausing to transcription elongation is regulated by the negative elongation factor (NELF) and the positive transcription elongation factor b (P-TEFb) through the phase separation mechanism (Fig. 4a).^51,65^ NELF functions as a transcriptional elongation repressor by stabilizing paused Pol II, whereas P-TEFb acts as a transcriptional elongation promoter by promoting the phosphorylation of NELF and Pol II, resulting in the release of Pol II from the paused state^66^ P-TEFb is a complex composed of the catalytic subunit cyclin-dependent kinase 9 (CDK9) and the regulatory subunit, cyclin T1 (CCNT1).^67–69^ The histidine-rich domain in CCNT1 can multivalently interact with the disordered CTD of Pol II, an event that promotes the P-TEFb phase to separate from the paused Pol II into a condensate.^51^ This allows CDK9 to phosphorylate not only the CTD of Pol II but also NELF. The phosphorylated CTD of Pol II interacts with various transcription elongation factors and RNA splicing factors to form elongation condensates and promote transcription.^41,70^ Further, the phosphorylation of NELF is essential for promoting the entry of Pol II into the elongation phase.^71^ NELF is a complex comprising four subunits, NELFA, NELFB, NELFC/D, and NELFE. Interestingly, NELF undergoes LLPS by the intrinsically disordered region of NELFA.^65^ However, under normal conditions, to promote the entry of Pol II into the elongation phase, NELF condensates are evenly distributed by phosphorylation at NELFA. When the cells are exposed to stress, inactivated P-TEFb cannot phosphorylate NELF, and the activated ZNF451, a SUMO E3 ligase, can SUMOylate NELF, which promotes the phase separation of NELF.^65^ Therefore, NELF can form condensates to inhibit the transcription of housekeeping genes and ensure cell survival.

In addition to these regulatory factors that can control the transcription condensates, the products of transcription, the RNAs, can also regulate the formation of transcription condensates by feedback mechanism (Fig. 4b).^35^ Jonathan et al. found that low levels of RNA at the regulatory DNA elements (super-enhancer, enhancer, and promoter) promote the formation of transcriptional condensates, whereas high levels of RNA from can dissolve the transcription condensates. As RNAs are usually negatively charged and proteins are usually positively charged in solution, they proposed that the interaction between proteins and RNAs in transcription condensates can be considered as a kind of poly-electrolyte.^35^ When the charges in the transcription condensates are equal, the opposite charge between RNA and protein promotes the formation of transcription condensates, whereas the negative charge caused by an increase in RNA levels and the repulsive charge between RNA and RNA causes the transcription condensates to dissolve.

### Phase separation in genome organization

**Fig. 5:**
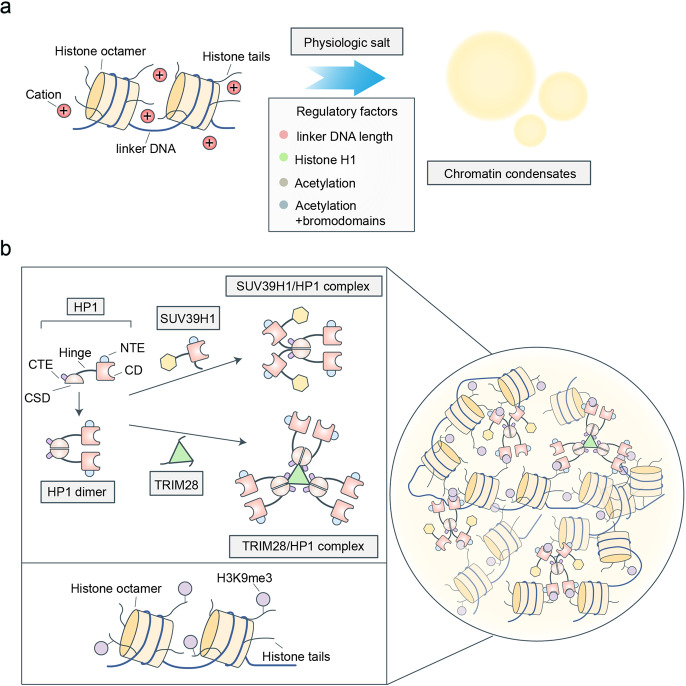
The function of LLPS in regulating genome organization. **a** The chromatin undergoes LLPS in physiologic salt, which is driven by the positively charged histone tails. Several factors can regulate the formation or properties of chromatin condensates, including the linker DNA length, histone H1, and histone acetylation. **b** The multivalent interactions between histone modifications and its readers have driven the formation of heterochromatin condensates. Heterochromatin protein 1 (HP1) contains a chromodomain (CD), a chromo shadow domain, and three disordered regions: N-terminal extension, hinge, and C-terminal extension. The HP1 dimer can interact with SUV39H1 (an H3K9me2/3 writer) and TRIM28 (an HP1 scaffolding protein) to form the SUV39H1/HP1 complex and TRIM28/HP1 complex, respectively. As these complexes contain multiple CDs that can interact with H3K9me2/3, they can phase-separate with the H3K9me2/3-marked nucleosome arrays to form condensates by multivalent interactions

Eukaryotic genomic DNA exists as chromatin in the nucleus.^72^ The basic functional unit of chromatin is the nucleosome, which contains ~146 bp of DNA wrapped around a histone octamer. A histone octamer is composed of two copies each of histones H2A, H2B, H3, and H4. The nucleosomes are connected by linker DNA and histone H1.^73^ Although we have a certain understanding of the composition of chromosomes, the specific mechanisms underlying chromatin organization and chromatin compartmentation are still not well understood. Recent results from Michael K. Rosen’s group indicate that the chromatin undergoes LLPS in physiological salt, a phenomenon that is driven by the positively charged histone tails (Fig. 5a).^74^ Further, they found that several factors can regulate the formation or properties of chromatin condensate. For example, the linker histone H1 promotes LLPS of chromatin and reduces the condensate dynamics, which is consistent with its role as a repressive chromatin architectural factor in cells. After hyperacetylation of the core histone tails, the chromatin condensates are dissolved, which facilitates the activation of transcription.^74^ In addition to chromatin, some special “readers,” which can recognize the corresponding histone modifications, are also involved in regulating chromatin compartmentation via LLPS.

Eukaryotic chromosomes contain various functional compartments, which are marked by specific histone modifications.^75^ Constitutive heterochromatin is a largely silent chromosome compartment, which is enriched with H3K9me2/3.^76^ The H3K9me3 reader protein is heterochromatin protein 1 (HP1), which contains a chromodomain (CD), a chromo shadow domain (CSD), and three disordered regions, i.e., the N-terminal extension (NTE), hinge, and C-terminal extension (CTE). The CD can bind to H3K9me3, whereas CSD contributes to the dimerization of HP1; the hinge region with patches of positively charged lysines is responsible for binding to DNA or RNA (Fig. 5b).^77^ Recently, Strom et al. found that the HP1 homolog in *Drosophila*, i.e., HP1a can form liquid-like condensates in vitro at higher concentrations under physiological conditions, which may mediate the heterochromatin domain formation in early *Drosophila* embryos.^22^ In mammals, there are three types of HP1 homologs, i.e, HP1α, HP1β, and HP1γ.^77^ Of these, only HP1α can undergo LLPS in vitro at high concentrations and low salt concentrations, which is driven by its disordered NTE and hinge region. Interestingly, over time, the HP1α condensates will reduce and exhibit gel-like properties.^24^ This is consistent with the function of HP1, which is to mediate gene silencing in the constitutive heterochromatin regions by inhibiting the binding of transcription factors to DNA. Both phosphorylation of NTE and the addition of nucleic acid can promote the formation of HP1α condensates. Additionally, when the lysine in the hinge region is mutated to an uncharged amino acid, the ability of HP1α to phase-separate is significantly reduced.^24^ These results indicate that the HP1α phase separation may be mediated by weak electrostatic interactions. In addition to electrostatic interactions, Wang et al. showed that the interactions between CD and H3K9me2/3 can also contribute to the LLPS of HP1 (Fig. 5b).^78^ The HP1 dimer can interact with SUV39H1 (an H3K9me2/3 writer) and TRIM28 (an HP1 scaffolding protein) to form the SUV39H1/HP1 complex and TRIM28/HP1 complex, respectively.^79–82^ As these complexes contain multiple CDs that can interact with H3K9me2/3, they can phase-separate with the H3K9me2/3-marked nucleosome arrays to form condensates by multivalent interactions. This review may provide a framework for researching the relationship between LLPS and epigenetics.

Overall, these evidences show that multivalence-driven LLPS plays an important role in genome organization.

### Phase separation and immune response

The human immune system comprises innate and adaptive immunity.^83^ T- and B-cells are the main cell types involved in adaptive immunity, and they have several specific and unique receptors. The numerous B lymphocytes consist of multitudinous B-cell receptors (BCRs), thus increasing the probability of encountering an antigen that binds specifically to a BCR. The process of clonal expansion of B cells, however, requires 3 to 5 days, which could grant the pathogen enough time to multiply and cause damage.^84^ Compared with adaptive immunity, innate immunity can be activated quickly after pathogen invasion. It is the first line of defense of the body against pathogen invasion until a sufficient number of antibodies are produced by B cells.^85^

**Fig. 6:**
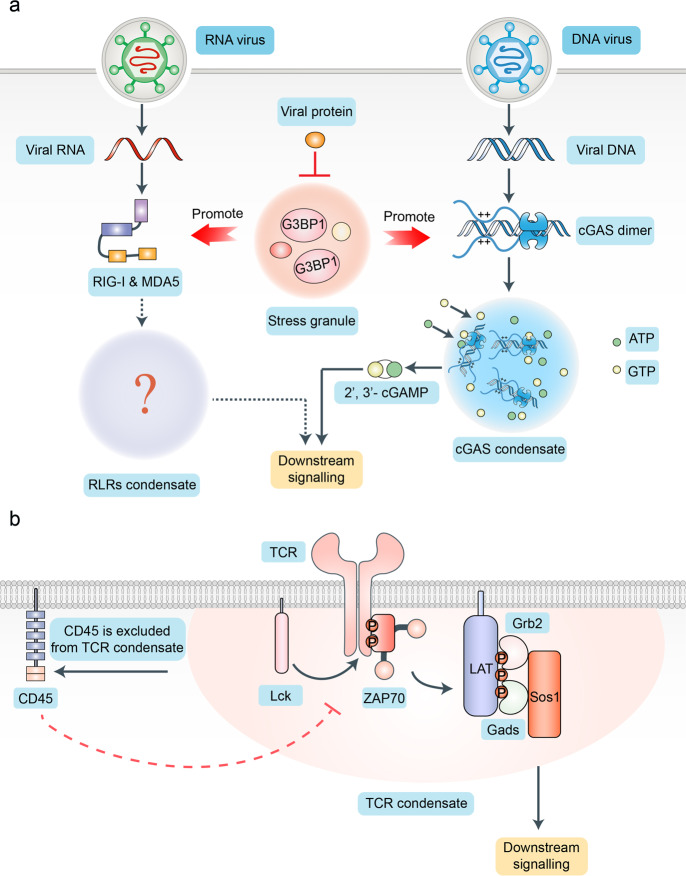
Phase separation and immune response. **a** LLPS is involved in innate immune responses. Cyclic GMP–AMP synthase (cGAS)- cytosolic DNA (such as viral DNA) condensates are formed by the multivalent interactions between cGAS and DNA via LLPS. The cGAS-DNA condensates may function as a reaction crucible, which can concentrate the reactants (ATPs and GTPs) and enzymes (activated cGAS) to efficiently produce cyclic GMP–AMP (cGAMP), which initiates anti-viral immune responses via the TBK1/IKK signaling pathway. Similarly, RLRs, such as RIG-I and MDA5, can also sense the viral nucleic acids (DNA or RNA), thereby initiating anti-viral immune responses via the TBK1/IKK signaling pathway. However, whether RLRs can phase-separate with nucleic acids to promote anti-viral immune responses is still not clear. Ras-GTPase-activating protein SH3 domain-binding protein 1 (G3BP1) is a positive regulator of innate immune responses (including RIG-I-mediated cellular anti-viral pathway and cGAS-STING pathway) and a stress granule core protein. SARS-CoV-2 N protein forms condensates that incorporate RNA and G3BP1, which suppress the interaction between G3BP1 and cGAS or RIG-I, thereby inhibiting the anti-viral immune responses of the host cells. **b** LLPS mediates T-cell receptor (TCR) signal transduction. After being phosphorylated by Lck, a kinase of the Src family, the cytoplasmic domains of TCR will recruit and activate the tyrosine kinase ZAP70. Thereafter, the multiple tyrosine residues of LAT, a transmembrane protein, are phosphorylated by activated ZAP70. These phosphorylated tyrosine residues recruit the SH2 and SH3 domain-containing protein GRB2, Gads, and Sos1, thereby activating T cells through several downstream signaling pathways, such as the MAPK pathway. These molecules can form condensates to exclude CD45, which can dephosphorylate the phosphorylated cytoplasmic domains of the TCRs to inhibit T-cell activation

In vertebrates, when pathogens invade, innate lymphocytes, such as macrophages and dendritic cells, recognize and bind to pathogen-associated molecular patterns (PAMPs) or danger-associated molecular patterns (DAMPs) through pattern-recognition receptors (PRRs), which induces the expression of pro-inflammatory factors, interferons, and hundreds of interferon-stimulated genes (ISGs).^86–90^ These effectors help the host to rapidly dispose off the invading pathogens. PAMPs are highly conserved molecular structures unique to a group of specific microbial pathogens and their products. The best-known examples of PAMPs include single-stranded or double-stranded RNA, DNA of viruses, and lipopolysaccharide (LPS) of Gram-negative bacteria. PAMPs do not exist in the host; therefore, they are regarded by the innate immune cells as molecular characteristics of infection. Currently, a variety of PRRs have been discovered. According to their localization in the cell, they can be divided into two categories: the first type of PRRs are located on the cell membrane, such as Toll-like receptors (TLRs);^91,92^ the second type of PRRs localizes in the cytoplasm of most cells, such as RIG-I-like receptors (RLRs) and cyclic GMP–AMP synthase (cGAS).^93^ The DNA sensor, cGAS, can catalyze the production of cyclic GMP–AMP (cGAMP) by using ATP and GTP.^94^ The adaptor protein stimulator of interferon genes (STING) will be activated by cGAMP, thereby inducing type I interferons.^95,96^ After being activated by cytosolic DNA (such as viral DNA), cGAS and DNA can form “foci” in the cytoplasm.^94^ We had not understood the specific molecular mechanism and function of these “foci” for a long time. A recent study by Du et al. revealed that the cGAS “foci” is formed by the multivalent interactions between cGAS and DNA via LLPS (Fig. 6a).^15^ Specifically, the disordered and positively charged cGAS N-terminal domain (NTD) and long DNA can promote the LLPS of cGAS-DNA by increasing valency (electrostatic interaction).^15^ The cGAS-DNA condensates may function as a reaction crucible, which can concentrate the reactants (ATPs and GTPs) and enzymes (activated cGAS) to efficiently produce cGAMP. Similarly, RLRs, such as RIG-I and MDA5, can also sense the viral nucleic acids (DNA or RNA), thereby initiating anti-viral immune responses via the TBK1/IKK signaling pathway.^97^ However, it is still not clear whether RLRs can phase-separate with nucleic acids to promote anti-viral immune responses. Besides, to escape detection of the immune system, viruses can inhibit the formation of these sensor condensates by manipulating some vital proteins, which can promote the phase separation or activation of sensors. For example, the Ras-GTPase-activating protein SH3 domain-binding protein 1 (G3BP1) is a positive regulator of innate immune responses (including the RIG-I-mediated cellular anti-viral pathway and cGAS-STING pathway) and a stress granule core protein.^98–100^ SARS-CoV-2 N protein forms condensates that incorporate RNA and G3BP1, which suppress the interaction between G3BP1 and cGAS or RIG-I, thereby inhibiting the host cell’s anti-viral immune response.^32^

Beyond innate immune responses, LLPS also mediates the signal transduction of adaptive immune responses (Fig. 6b). T-cell receptor (TCR) signal transduction is vital for T-cell activation.^101,102^ After being phosphorylated by Lck, a kinase of the Src family, the cytoplasmic domains of TCR will recruit and activate the tyrosine kinase ZAP70.^103^ Multiple tyrosine residues of LAT, a transmembrane protein, are phosphorylated by the activated ZAP70. These phosphorylated tyrosine residues recruit the SH2 and SH3 domain-containing proteins GRB2, Gads, and Sos1, thereby activating T cells through several downstream signaling pathways, such as the MAPK pathway.^101,104^ Interestingly, a transmembrane phosphatase, CD45, can dephosphorylate the phosphorylated cytoplasmic domains of the TCR to inhibit T-cell activation.^105^ The underlying mechanisms of the regulation of the balance of opposing functions between CD45 and Lck are unclear. Recent advances in LLPS show that the T-cell receptor (TCR) signaling cluster can phase-separate into a condensate, which is driven by the multivalent binding of SH3 and SH2 domains to their cognate motifs.^14^ Moreover, the TCR signal transduction inhibitor, CD45, is excluded from the condensates to ensure T-cell activation.

Overall, LLPS not only provides a useful new framework to understand the complex immune response but also presents exciting new avenues for therapeutic interventions by modulating the formation of these immune response condensates.

### Phase separation and neuronal synaptic signaling

Synapses are the sites where two neurons physically connect and communicate with each other, and they are the most basic unit of the brain network.^106^ Each synapse is formed by thousands of proteins and can change its composition and signal processing

capacities in response to various stimuli. Thus, synapses are dynamic micro-computational devices. Recent studies revealed that the LLPS mediates the formation of pre- and postsynaptic density signaling assemblies.^107^

Zeng et al.^16,17^ provided the first hint suggesting that postsynaptic density (PSD) may be formed by the the interaction between PSD-95 and SynGAP via phase separation. SynGAP and PSD-95 are two very abundant proteins existing at a near stoichiometric ratio in PSD.^108^ PSD-95 and its two homologous, PSD-93 and SAP102, are central scaffolding proteins in PSD, orchestrating multiple signaling cascades, as well as shaping the basic architecture of PSD.^109–111^ SynGAP, which catalyzes the conversion of small G proteins such as Ras and RAP from their GTP-bound forms to the GDP-bound forms, serves as an inhibitory factor for synaptic activities.^112^ SynGAP forms a parallel coiled-coil trimer capable of binding to multiple copies of PSD-95. This multivalent SynGAP/PSD-95 interaction leads to the formation of LLPS, both in vitro and in living cells. Importantly, the formation of SynGAP/PSD-95 condensates is vital for SynGAP stabilization in PSD and for preventing neurons from hyper-excitation.^16^ Moreover, by using reconstituted PSD system, they showed that transmembrane AMPAR regulatory proteins (TARPs), which are a family of auxiliary subunits of AMPARs critical for the trafficking and transmission of the ion channel in synapses, are clustered in the PSD condensates via phase separation.^113^ Importantly, charge neutralization mutations in TARP C-terminal tail Arg-rich motif weakened TARP’s condensation with PSD-95 and impaired TARP-mediated AMPAR synaptic transmission in mice hippocampal neurons.^113^ Therefore, LLPS-mediated PSD assembly formation and regulation are linked with the physiological functions of synapses.

In addition to PSD signaling assemblies, LLPS also play a crucial role in presynaptic density signaling assemblies. Wu et al. recently demonstrated that the multivalent interaction between RIM and RIM-BP (RIM and RIM-BP are scaffold proteins in presynaptic densities) drives the formation of RIM/RIM-BP condensates.^114^ Importantly, the cytosolic tail of voltage-gated Ca^2+^ channels (VGCCs) can be recruited to the RIM/RIM-BP condensates via direct binding of the Ca^2+^ channel tail to both RIM and RIM-BP, resulting in a massive enrichment of the channel. This is consistent with the concept that fast and accurate neurotransmitter release is supported by both the density of clustered VGCCs on presynaptic plasma membranes and the proximity of the clustered VGCCs to calcium sensors at the synaptic vesicles (SV) fusion sites.^115,116^ In addition, Milovanovic et al. showed that synapsin, an abundant synaptic vesicle–associated protein, organizes the formation of vesicle clusters by LLPS.^117^ And it is important for maintaining the stability of the reserve pool SV and possibly priming these vesicles for being transported to the release sites upon arrival of action potentials.

## Pathological functions of LLPS

### Phase separation in neurodegenerative disease

**Fig. 7:**
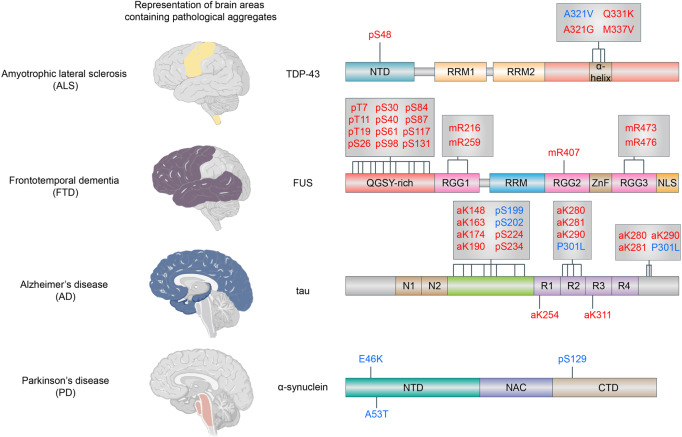
Phase separation in neurodegenerative disease. Schematic representation of brain areas containing pathological aggregates of four kinds of neurodegenerative diseases. This figure also summarizes disease-associated mutations and PTMs that decrease (red) or enhance (blue) LLPS compared with unmodified TDP-43, FUS, a-synuclein, or tau, as indicated. NTD: N-terminal domain, RRM: RNA-recognition motif, QGSY-rich: rich in glutamine, glycine, serine, and tyrosine, RGG: arginine/glycine-rich; ZnF, zinc finger; NLS, nuclear localization signal; NAC, non-Ab component of AD plaque; N1–2, polypeptide sequences encoded by exons 2 and 3; PRR, proline-rich regions; R1–4, microtubule-binding domains encoded by exons 9–12; p, phosphorylation; m, methylation; a, acetylation

Under physiological conditions, LLPS is indeed vital for wide range of biological processes and systems. However, an increasing set of proteins that can physiologically undergo LLPS are also found in pathological aggregates.^118^ These results indicate that pathological protein aggregates originate from an aberrant phase separation. Notably, protein aggregates are main hallmark of several neurodegenerative diseases, including amyotrophic lateral sclerosis (ALS), frontotemporal dementia (FTD), Alzheimer’s disease (AD), and Parkinson’s disease (PD).^119,120^ The transition from reversible dynamic LLPS to an irreversible aggregation state has been shown for TAR DNA-binding protein 43 (TDP-43), fused in sarcoma (FUS), tau, and *α*-synuclein, which are found aggregated in affected neurons of patients with ALS, FTD, AD and PD, respectively.^118,121–124^ These processes are regulated by disease-associated mutations and post-translational modifications (PTMs). Here, we review the intrinsic driving force and modulators (disease-associated mutations and PTMs) of LLPS in these four neurodegenerative disease-associated proteins (Fig. 7).

LLPS of TDP-43 is mediated by aromatic, dipolar, and positively charged (arginine) residues in the IDR, as well as a transiently formed a-helix.^122,125^ Most ALS-associated mutations in the helix decrease LLPS, with a rare exception of A321V, which enhances it.^125^ NTD-mediated oligomerization contributes to LLPS of TDP-43 and an oligomerization-disrupting mimic of phosphorylation at S48 decreases it.^126,127^ Interactions between arginines (positively charged) in one arginine/glycine-rich (RGG) motif and tyrosines (aromatic) in the IDR of FUS are implicated in condensate formation.^128^ Phosphorylation and methylation on multiple positions on the FUS sequence decrease LLPS.^128,129^ *α*-synuclein LLPS is driven by electrostatic interactions in the amphiphilic N-terminal domain and hydrophobic interactions between the non-Ab component of AD plaque (NAC) domains.^123^ PD-associated mutations (E46K, A53T) as well as a phosphorylation in S129 increase LLPS.^123^ Tau LLPS is driven by electrostatic interactions between the negatively charged N-terminal and positively charged C-terminal regions of the protein.^130,131^ Phosphorylation (S199, S202) and a mutation in the P301L have been shown to increase LLPS while acetylation in different domains are correlated with a decrease in LLPS.^132^

The effect of different mutations on the phase separation behavior of proteins is not yet fully understood. However, it is possible that mutations that lower the saturation concentration could trap the protein in a condensed system, favoring the transition from reversible dynamic LLPS to an irreversible aggregation state over time. Additionally, mutations may alter the binding to cellular LLPS modulators. For example, FUS mutations indirectly suppress its physiological LLPS behavior and promote aggregation by preventing the interaction with its nuclear import receptor Transportin-1, which presents a chaperone/disaggregase activity in vitro and in vivo.^28,29,128,133^ As for the regulatory effect of different PTMs on the phase separation behavior, the presence of some PTMs might trigger abnormal transitions by preventing the physiologically regulating PTMs in the same residue(s) that either suppress or maintain LLPS under physiological conditions. More studies on the regulation and the role of these PTMs in physiological and pathological LLPS are necessary for us to development new drugs.

### LLPS in cancer

Cancer is considered a gene mutation or dysregulated gene transcription disease. Typically, mutations in proto-oncogenes lead to increased gene products or increased activity of the products, thereby causing excessive cell proliferation to form tumors.^134^ Similarly, mutant cancer suppressor proteins may have reduced biological activities, which can also promote the formation of tumors.^135,136^ Although remarkable progress in recent years has been made in the identification of specific mutations in proto-oncogenes and tumor-suppressor genes that could cause cancer, we still do not completely understand the specific mechanisms underlying why these mutations cause cancer. Recent advances in LLPS may provide a new framework to understand the relationship between mutation and cancer.

**Fig. 8:**
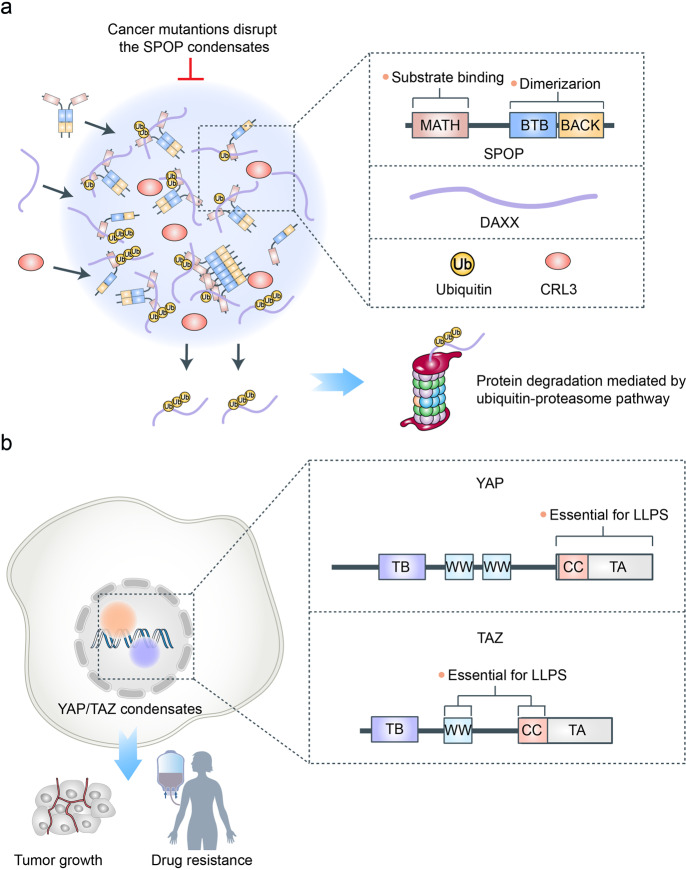
LLPS in cancer. **a** Speckle-type POZ protein (SPOP) can phase-separate with its substrates and cullin3-RING ubiquitin ligase to form condensates, which promote the degradation of its substrates via the ubiquitin-proteasome system. SPOP consists of a substrate-binding meprin and TRAF homology (MATH) domain and two dimerization domains, BTB and BACK. The self-association by two dimerization domains and the multievent interactions between MATH domain and substrates are necessary for the phase separation of SPOP. Cancer-associated mutations in the MATH domain disrupt the formation of SPOP condensate by preventing the interaction between substrates and SPOP. **b** The transcriptional coactivators Yes-associated protein (YAP) and transcriptional coactivator with PDZ-binding motif (TAZ) can activate the transcription of various genes via LLPS. The Hippo signaling pathway can inhibit the formation of YAP/TAZ condensates. However, the Hippo signaling pathway is inactivated in many cancers. Therefore, the accumulation of TAZ and YAP can largely activate the transcription of proto-oncogenes via phase separation, promoting cell proliferation and anti-PD-1 immunotherapy resistance via LLPS. The intrinsically disordered TA and CC domains are essential for the formation of YAP condensates, whereas the CC and WW domains are vital for TAZ phase separation

A mutation in the Speckle-type POZ protein (SPOP), a tumor-suppressor protein, can lead to the formation of many solid tumors, including prostate, gastric and colorectal cancers.^137,138^ SPOP functions as a substrate adaptor of a cullin3-RING ubiquitin ligase to promote the degradation of its substrates via the ubiquitin-proteasome system.^139–142^ The substrates of SPOP are various proto-oncogenic proteins, such as androgen receptor and death-domain-associated protein.^143–145^ The accumulation of these proteins can oncogenically transform sensitive cell types. Recently, a study revealed that these substrates can phase-separate with SPOP to form condensates in vitro and co-localize in liquid nuclear organelles in cells.^146^ The SPOP condensates promote the ubiquitination of its substrates. SPOP consists of a substrate-binding meprin, a TRAF homology (MATH) domain, and two dimerization domains, BTB and BACK (Fig. 8a).^147^ The self-association by two dimerization domains and the multivalent interactions between the MATH domain and substrates are necessary for the phase separation of SPOP. Moreover, cancer-associated mutations in the MATH domain disrupt the formation of the SPOP condensate by preventing the interaction between substrates and SPOP. The accumulation of oncogenic SPOP substrate proteins causes cancer. Similarly, mutant SHP2 (a non-receptor protein tyrosine phosphatase) can recruit and activate wildtype SHP2 in LLPS to promote MAPK activation, thereby promoting tumorigenesis.^148^ In addition to SPOP and SHP2, AKAP95, a nuclear protein that plays an important role in supporting tumorigenesis through splice regulation, also forms phase-separated and liquid-like condensates.^149^ Studies have revealed that mutations of key residues to different amino acids perturb AKAP95 condensation in opposite directions. Importantly, the activity of AKAP95 in splice regulation is abolished by disruption of condensation, significantly impaired by hardening of condensates, and regained by substituting its condensation-mediating region with other condensation-mediating regions from irrelevant proteins. Moreover, the abilities of AKAP95 in regulating gene expression and supporting tumorigenesis require AKAP95 to form condensates with proper liquidity and dynamicity.^149^ These results link phase separation to tumorigenesis and may provide opportunities for therapeutic interventions of cancer.

Beyond gene mutation, dysregulation of transcription is another hallmark of cancer.^150^ As mentioned above, transcriptional coactivators play a vital role in the regulation of transcription via LLPS. Indeed, several transcriptional coactivators that regulate tumorigenesis has been found to undergo LLPS, such as the Hippo pathway downstream effectors, the transcriptional coactivators Yes-associated protein (YAP), and the transcriptional coactivator with PDZ-binding motif (TAZ).^151,152^ YAP and TAZ can activate the transcription of various genes that regulate cell proliferation, organ size, and tumorigenesis.^153–156^ In normal cells, a variety of signals, such as hyperosmotic stress, cell-cell contact, and cell polarity, can activate the Hippo pathway to inhibit YAP and TAZ, thereby repressing the transcription of these genes.^157^ However, the Hippo signaling pathway is inactivated in many cancers.^158^ Therefore, the accumulation of TAZ and YAP promotes the initiation and growth of cancer cells. In addition to their functions, YAP and TAZ also have similar structures.^159^ They both contain the WW domains (YAP has two WW domains; TAZ has one WW domain), a TEAD-binding (TB) domain, a coiled-coil (CC) domain, and a transcription activation (TA) domain.^160^ Recent studies found that LLPS mediates the transcriptional activation induced by YAP/TAZ.^151,152,161^ In cancer cells, the YAP and TAZ condensates are involved in promoting cell proliferation and anti-PD-1 immunotherapy resistance.^161^ Besides, the YAP condensates are enriched in accessible chromatin domains organized as super enhancers, whereas the TAZ condensates contain DNA-binding cofactor TEAD4, coactivators BRD4 and MED1, and CDK9. Moreover, the intrinsically disordered TA and CC domains are essential for the formation of the YAP condensates, whereas the CC and WW domains are vital for the TAZ phase separation (Fig. 8b). The formation of the nuclear YAP and TAZ condensates is negatively regulated by Hippo signaling through LATS-mediated phosphorylation.

The emergence of LLPS provides a novel approach to target intractable and undruggable proteins, for example, it was recently reported that LLPS of disease-associated SHP2 mutants could be specifically attenuated by SHP2 allosteric inhibitors.^148^ Moreover, LLPS of cancer-associated SRC-1, a previously known transcriptional coactivator for nuclear hormone receptors, could be selectively disrupted by the treatment of an anti-HIV drug elvitegravir (EVG).^162^ More specific drugs that inhibit the formation of such aberrant condensates are potential new therapeutic cancer treatments.

### LLPS in SARS-CoV-2 infection

Infectious diseases are not only one of the most common diseases in humans, but also one of the main diseases that cause human deaths.^163^ The pathogens that cause infections mainly include parasites (such as trypanosoma, plasmodium, and toxoplasma) and other pathogenic microorganisms (including various viruses, bacteria, fungi, and mycoplasma).^164,165^ To eliminate pathogen infection, humans have evolved a very efficient and widely adapted immune system. However, pathogens have also formed a variety of immune escape mechanisms during its evolution. In the past few decades, although there has been remarkable progress in our understanding of the relationship between pathogen infection and the human anti-infection immune response, we still know little regarding the specific mechanisms underlying these complex processes. Recently, there is emerging evidence that LLPS is involved in viral diseases and the anti-viral infection immune response.^15,32,34,36,38,166–171^ It may thus provide a new framework to interpret and understand the mechanisms underlying infectious diseases and create potential new avenues for treatment.

SARS-CoV-2, a novel coronavirus, has caused the coronavirus disease 2019 global pandemic.^172^ Following the identification of the first case in China, there have been numerous studies that have been conducted, which provide a better understanding of this novel virus.^173–177^ After invading the host cell, the ~30-kb-positive-sense single-stranded RNA genome is released for viral genome replication, and translation of various proteins, including non-structural and structural proteins. There are four main structural proteins in SARS-CoV-2, including the membrane (M), envelope (E), nucleocapsid (N), and spike (S) proteins.^178,179^ Of these, the N protein is involved in the transcription of the viral genome, replication, and packaging.^180–183^ Emerging evidence shows that the N protein performs these functions via the LLPS mechanism.^32,36,38,170,171^

**Fig. 9:**
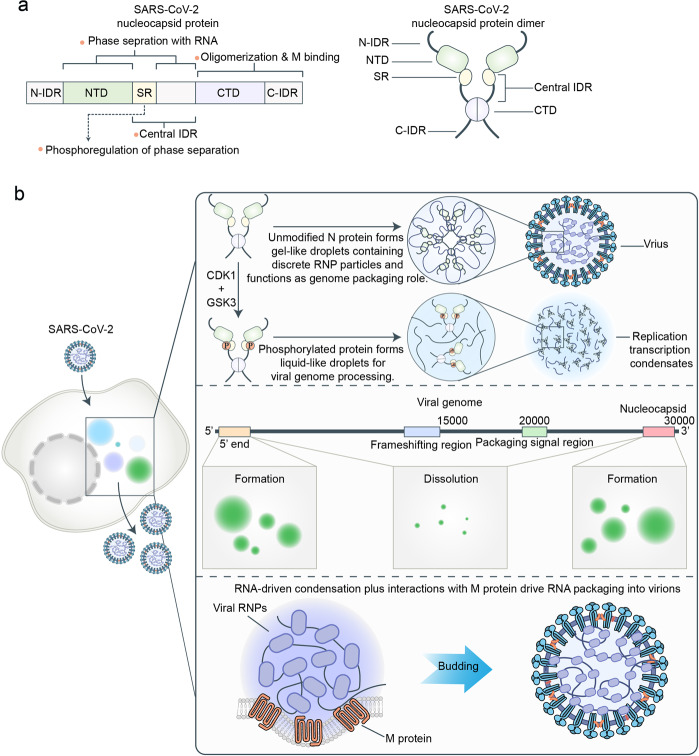
LLPS in SARS-CoV-2 infection. **a** Nucleocapsid (N) protein contains an N-terminal intrinsically disordered region (N-IDR), a structured N-terminal domain (NTD), a central intrinsically disordered region (central-IDR), a structured C-terminal domain (CTD), and a C-terminal intrinsically disordered region (C-IDR). Of these, NTD and central-IDR play an important role in the phase separation of N protein with RNA. **b** LLPS plays an important role in SARS-CoV-2 infection. The central-IDR contains a conserved serine-arginine (SR)-rich sequence. Unmodified N protein forms gel-like condensates containing discrete ribonucleoprotein (RNP), which is conducive to its function of genome packaging roles. After being phosphorylated by cyclin-dependent kinase-1 (CDK1) and glycogen synthase kinase-3 (GSK3) in the SR region, N protein forms liquid-like condensates for viral genome processing. Viral RNA sequence and structure in specific genomic regions are also involved in the regulation of the N protein phase separation. The specific regions of the viral RNA genome, including a region spanning the 5’ end (first 1000 nt) and a region-encoded N protein at the 3᾽ end, can promote the phase separation of N proteins, whereas most regions in the genome facilitate the dissolution of N protein condensates. The viral membrane (M) protein can independently form condensates with N protein. During the late stages of SARS-CoV-2 infection, the viral RNP condensates will interact with the soluble CTD of M protein to form two-layered condensates, which promote the SARS-CoV-2 virion assembly

N proteins can phase-separate with RNA to form condensates at body temperature of humans and are enhanced by Zn^2+^.^34,36^ The N protein consists of five parts from the N-terminal to the C-terminal, including an N-terminal intrinsically disordered region (N-IDR), a structured N-terminal domain (NTD), a central intrinsically disordered region (central-IDR), a structured C-terminal domain (CTD), and a C-terminal intrinsically disordered region (C-IDR) (Fig. 9a).^184^ Of these, the NTD and central-IDR play an important role in the phase separation of the N protein with RNA.^36^ Moreover, the central-IDR contains a conserved serine-arginine (SR)-rich sequence.^185,186^ Unmodified N protein forms gel-like condensates containing discrete ribonucleoprotein (RNP), which is conducive for its genome packaging roles. After being phosphorylated by cyclin-dependent kinase-1 (CDK1) and glycogen synthase kinase-3 (GSK3) in the SR region, the N protein forms liquid-like condensates for viral genome processing (Fig. 9b).^38^ Therefore, phosphorylation of the SR region regulates the distinct functions of the N protein in genome packaging and processing by transforming the material properties of the N protein condensates from gel-like to liquid-like. In addition to phosphorylation, the viral RNA sequence and structure in specific genomic regions are also involved in the regulation of the N protein phase separation. Recent evidence from Christiane et al. indicates that the specific regions of the viral RNA genome, including a region spanning the 5᾽-end (first 1000 nt) and a region encoding the 3᾽-end of the N protein can promote the phase separation of N protein, whereas most other regions in the genome facilitate the dissolution of N protein condensates (Fig. 9b).^34^ The balance between phase separation-promoting and solubilizing regions in the viral RNA genome may be beneficial for N proteins to package the viral genome. In addition to RNA, the transmembrane M protein can also independently form condensates with N protein (Fig. 9b).^32^ The interactions between the soluble CTD of M protein and the C-IDR of N protein mediates this process. Interestingly, when adding RNA to the condensates, which are formed by M protein and N protein, it will form two-layered condensates with a central core of N protein + RNA surrounded by a shell of N protein + M protein.^32^ These results suggest that during the late stages of SARS-CoV-2 infection, the viral RNP condensates will interact with the soluble CTD of the M protein to form a two-layered condensate, which promotes the SARS-CoV-2 virion assembly.

Emerging evidence indicates that in addition to SARS-CoV-2, many viruses can form viral replication condensates via LLPS.^166–168^ Overall, these exciting studies provide a new framework for understanding the mechanisms underlying the virus’s replication in host cells and may be useful in developing anti-viral drugs.

## Conclusions and perspectives

Over the past decade, LLPS has become an attractive area of research. Despite some breakthroughs, our understanding of the LLPS is still in its infancy. An increasing number of questions have also emerged. For example, what is the underlying mechanism in the regulation of biomolecular condensates by their material properties? How disease-associated mutations or PTMs regulate the physical properties of condensates? And, how to regulate LLPS to achieve the desired therapeutic effect still need to be explored.

Importantly, more quantitative tools or approaches need to be developed and applied to LLPS research. FRAP is extensively used to prove the process of liquid phase separation. However, it must be pointed out that in different studies, FARP recovery time rates for the same molecule can range from less than one minute to several minutes, suggesting that FARP is not the gold standard for confirming LLPS. Moreover, in the cell-free reconstitution assays cited in many research articles, the conditions for molecular phase separation deviate greatly from physiological conditions, including high salt concentration, high or low pH buffer, and high protein concentration. As a result, the conclusion of the in vitro experiment is not consistent with the true phase separation condition or function in the cell.

Overall, although the field of LLPS is young and fast-growing, this mechanism has undoubtedly revolutionized our understanding of various biological activities and disease conditions. It is expected that basic research on LLPS and human diseases will continue to improve and translate into clinical practice.
